# Correction: Intraocular pressure, primary open-angle glaucoma and the risk of retinal vein occlusion: a Mendelian randomization mediation analysis

**DOI:** 10.1038/s41433-024-03340-6

**Published:** 2024-09-13

**Authors:** Andreas Katsimpris, Sebastian-Edgar Baumeister, Nafsika Voulgari, Hansjörg Baurecht, Stylianos Kandarakis, Michael Nolde

**Affiliations:** 1 Princess Alexandra Eye Pavilion, University of Edinburgh, Edinburgh, UK; 2 Institute of Health Services Research in Dentistry, University of Münster, Münster, Germany; 3 Department of Epidemiology and Preventive Medicine, University of Regensburg, Regensburg, Germany; 4 First Department of Ophthalmology, National and Kapodistrian University of Athens, Athens, Greece

**Keywords:** Risk factors, Retinal diseases

Correction to: *Eye* 10.1038/s41433-024-03303-x, published online 15 August 2024

The first and last name of the author Hansjörg Baurecht were unfortunately interchanged. The original article has been corrected.

